# A novel prediction model of the risk of pancreatic cancer among diabetes patients using multiple clinical data and machine learning

**DOI:** 10.1002/cam4.6547

**Published:** 2023-09-22

**Authors:** Shih‐Min Chen, Phan Thanh Phuc, Phung‐Anh Nguyen, Whitney Burton, Shwu‐Jiuan Lin, Weei‐Chin Lin, Christine Y. Lu, Min‐Huei Hsu, Chi‐Tsun Cheng, Jason C. Hsu

**Affiliations:** ^1^ School of Pharmacy Taipei Medical University Taipei Taiwan; ^2^ International Ph.D. Program in Biotech and Healthcare Management, College of Management Taipei Medical University Taipei Taiwan; ^3^ Clinical Data Center, Office of Data Science Taipei Medical University Taipei Taiwan; ^4^ Clinical Big Data Research Center Taipei Medical University Hospital, Taipei Medical University Taipei Taiwan; ^5^ Research Center of Health Care Industry Data Science, College of Management Taipei Medical University Taipei Taiwan; ^6^ Section of Hematology/Oncology, Department of Medicine and Department of Molecular and Cellular Biology Baylor College of Medicine Houston Texas USA; ^7^ Department of Population Medicine Harvard Medical School and Harvard Pilgrim Health Care Institute Boston Massachusetts USA; ^8^ Kolling Institute, Faculty of Medicine and Health The University of Sydney and the Northern Sydney Local Health District Sydney New South Wales Australia; ^9^ School of Pharmacy, Faculty of Medicine and Health The University of Sydney Sydney New South Wales Australia; ^10^ Graduate Institute of Data Science, College of Management Taipei Medical University Taipei Taiwan

**Keywords:** artificial intelligence, diabetes, machine learning, pancreatic cancer, prediction model, Taipei Medical University Clinical Research Database (TMUCRD)

## Abstract

**Introduction:**

Pancreatic cancer is associated with poor prognosis. Considering the increased global incidence of diabetes cases and that individuals with diabetes are considered a high‐risk subpopulation for pancreatic cancer, it is critical to detect the risk of pancreatic cancer within populations of person living = with diabetes. This study aimed to develop a novel prediction model for pancreatic cancer risk among patients with diabetes, using = a real‐world database containing clinical features and employing numerous artificial intelligent approach algorithms.

**Methods:**

This retrospective observational study analyzed data on patients with Type 2 diabetes from a multisite Taiwanese EMR database between 2009 and 2019. Predictors were selected in accordance with the literature review and clinical perspectives. The prediction models were constructed using machine learning algorithms such as logistic regression, linear discriminant analysis, gradient boosting machine, and random forest.

**Results:**

The cohort consisted of 66,384 patients. The Linear Discriminant Analysis (LDA) model generated the highest AUROC of 0.9073, followed by the Voting Ensemble and Gradient Boosting machine models. LDA, the best model, exhibited an accuracy of 84.03%, a sensitivity of 0.8611, and a specificity of 0.8403. The most significant predictors identified for pancreatic cancer risk were glucose, glycated hemoglobin, hyperlipidemia comorbidity, antidiabetic drug use, and lipid‐modifying drug use.

**Conclusion:**

This study successfully developed a highly accurate 4‐year risk model for pancreatic cancer in patients with diabetes using real‐world clinical data and multiple machine‐learning algorithms. Potentially, our predictors offer an opportunity to identify pancreatic cancer early and thus increase prevention and invention windows to impact survival in diabetic patients.

## Graphical abstract

To predict the risk of pancreatic cancer in patients with diabetes is crucial; the study aims to establish a novel prediction model for pancreatic cancer risk among patients with diabetes using real‐world clinical data.

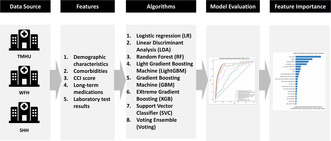

## 1 INTRODUCTION

Pancreatic cancer has one of the worst prognoses among all cancer types. Its pathophysiology makes it exceptionally difficult for early‐stage detection. Due to a lack of effective screening and diagnostic tools, most pancreatic cancer cases are diagnosed when the tumor has already reached a locally advanced or metastatic stage. Even among patients who undertake surgical interventions, poor prognosis is still high. Of the 10%–20% of pancreatic cancer patients who undergo surgical resection after diagnosis, only about 20% have a 5‐year survival rate.^1^, ^2^ Comparatively, patients without surgical resection have far worse outcomes with a 5‐year survival rate of <5%.^3^ Previous research highlights that the significant risk factors for pancreatic cancer include age, gender, race, a family history of inheritance, smoking, drinking, obesity, chronic pancreatitis, hepatitis, and persons living with human immunodeficiency virus (PLWH).^4^, ^5^, ^6^ Additionally, patients with diabetes mellitus (DM) have an elevated risk for pancreatic cancer.^6^, ^7^, ^8^, ^9^

Regarding the correlation between diabetes and pancreatic cancer, previous publications observed that up to 85% of pancreatic cancer patients have diabetes at the time of the cancer diagnosis.^10^ The increased risk of pancreatic cancer is two to three times higher for patients with diabetes than patients without diabetes.^11^, ^12^, ^13^ Both newly diagnosed and long‐term diabetes patients have clear associations with pancreatic cancer.^14^, ^15^, ^16^ An additional concern is that DM is associated with a high risk of mortality for cancer patients. There are also studies identifying insulin resistance and hyperinsulinemia as factors related to the risk of pancreatic cancer among patients with long‐term diabetes.^17^

Previously, many scholars developed prediction models for the risk of pancreatic cancer in patients with diabetes. One, in particular, Dong et al. highlighted the use of traditional logistic regression (LR) model and found eight important predictive factors, including the age of onset of diabetes, body mass index (BMI), hepatitis B virus (HBV), total bilirubin (TBIL), alanine aminotransferase (ALT), creatinine (Cr), apolipoprotein A1 (APO‐A1), and white blood cells (WBCs).^18^ This research established a prediction model with an area under the receiver operating characteristics curve (AUROC) of 0.81517. Hsieh et al. applied data from the Taiwan National Health Insurance database and developed prediction models using both traditional LR and deep‐learning Artificial Neural Network (ANN) algorithms, with results showing that the traditional LR model (AUROC = 0.727) was more accurate than the ANN model.^2^ Boursi et al. (2022) used data from The Health Improvement Network (THIN) database of the UK and used a traditional LR to establish a prediction model for the risk of pancreatic cancer within 3 years for patients with pre‐diabetes, with an AUROC of 0.71 and important predictive factors including age, BMI, total cholesterol, proton pump inhibitor use, ALT, low‐density lipoprotein, alkaline phosphatase, etc.^19^

Diabetes and pancreatic cancer have a high degree of association. Previous studies have established pancreatic cancer risk prediction models for patients with diabetes. However, they lacked the use of complete clinical data types and multivariate machine learning algorithms, resulting in unsatisfactory model performance. This study aimed to comprehensively identify features that influence the occurrence of pancreatic cancer in diabetic patient populations and develop a more accurate AI‐based prediction model for personalized pancreatic cancer risk assessment.

## 2 METHODS

### 2.1 Study design and data source

This retrospective study was conducted utilizing archived data from the Taipei Medical University Clinical Research Database (TMUCRD), which gathers different types of electronic medical records (EMR) from three medical centers located in Taiwan—TMU Hospital (TMUH), Wan‐Fang Hospital (WFH), and Shuang‐Ho Hospital (SHH). The TMUCRD collects structured and unstructured data, such as patient demographic information, medical visits, test reports, diagnostic results, treatment steps, surgeries, medication information, physician notes, radiology, and pathology reports. The TMUCRD covers data from 1998 to 2022 and includes 12 categories, 66 tables, and 2491 fields. Each record session is linked to the others. From 1998 to 2021, the database accumulated medical information on 4,125,097 patients. The database was linked to the Taiwan Cancer Register, a national registrar to obtain more information about cancer treatment. To obtain accurate date of death and cause of death information for each deceased patient in the TMUCRD was also linked to death registration records in Taiwan. All data were anonymized before the analysis.

Real‐world evidence (RWD) can be obtained from multiple sources, including electronic health records (EHRs), medical claims, billing data, and insurance data. Additionally, data from product and disease registries, as well as patient‐generated data from in‐home‐use settings, contribute to the generation of RWD. Furthermore, data collected from other sources such as mobile devices can also provide valuable information that helps assess and monitor health status.^20^, ^21^

Our database, the Taipei Medical University Clinical Research Database (TMUCRD), collects diverse electronic health records (EHRs) from three prominent medical centers in Taiwan: TMU Hospital (TMUH), Wan‐Fang Hospital (WFH), and Shuang‐Ho Hospital (SHH). Based on this information, it is reasonable to consider our database as a form of real‐world data (RWD). The TMUCRD provides a valuable resource for conducting clinical research, enabling the analysis of patient health information and the evaluation of healthcare practices in real‐world settings.

### 2.2 Population

**FIGURE 1:**
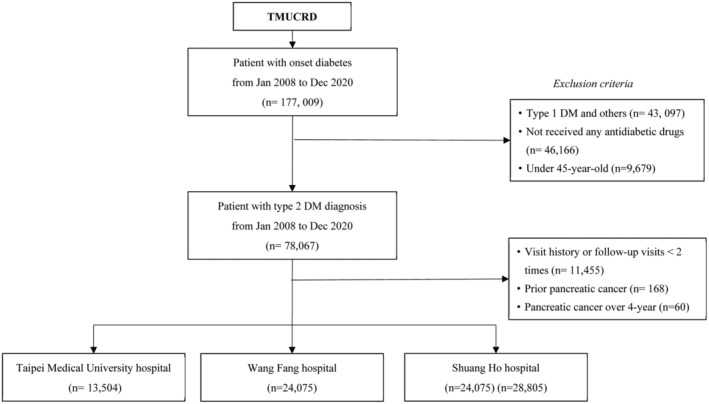
Population selection process flowchart.

This study recruited individuals diagnosed with diabetes (International Classification of Diseases (ICD)‐9: 250; ICD‐10: E11). The initial selection included records from January 1, 2009, to December 31, 2019, of individuals who utilized services within the outpatient department (OPD) or were admitted to the inpatient department (IPD). We excluded individuals under the age of 40 years, patients with Type I DM (T1DM), and those who had previously been diagnosed with pancreatic cancer (ICD‐O‐3: C25) prior to a Type 2 DM (T2DM) diagnosis. Patients without visit history and those who received no antidiabetic medications (Anatomical Therapeutic Chemical (ATC) code: A10) for DM were also excluded. Subsequently, 66,384 records from the TMURCD were used for this study. This included 13,504 patients from TMUH, 24,075 patients from WFH, and 28,805 patients from SHH. Figure 1 shows our population selection process.

### 2.3 Outcome measurement

The index date for this study was characterized as the date when antidiabetic medications were prescribed. The study's aim was to define any occurrence of pancreatic cancer within 4 years following this index date.^22^ Patients with pancreatic cancer were identified using data from the TMUCRD with the ICD‐O‐3 code C25. The participants were amended at a loss to follow‐up, mortality date, or at the end of the study, on December 31, 2019.

### 2.4 Predictors

We deployed predictors affiliated with the outcomes in accordance with a literature review and clinicians' recommendations.^17^, ^18^, ^23^, ^24^, ^25^ The predictors included diagnoses, medications, and laboratory tests from outpatient or admission datasets. The particular predictors were as follows:

1. Demographic characteristics (i.e., gender, age, and body mass index (BMI))
2. Comorbidities before the prescription date of antidiabetic drugs (i.e., cardiovascular, chronic obstructive pulmonary, and rheumatic diseases) and the Charlson comorbidity index (CCI) score
3. Long‐term medications (i.e., antacids, gastroesophageal reflux disease (GORD), and gastrointestinal disorder agents) are prescribed during the 6 months before a prescription for an antidiabetic drug
4. Laboratory test results (i.e., glycated hemoglobin (HbA1c), glucose AC, and albumin) within 12 months before prescription of an antidiabetic drug.

Median imputation was applied for missing continuous predictors.

### 2.5 Statistical analysis

Descriptive analyses of the study population, including the frequency (%) and mean (standard deviation [SD]) for categorical and numerical variables, were conducted. The univariate analysis investigated significant correlations between factors and the outcome variable. A Logistic Regression (LR) with univariate and multivariate analytical methods was used to estimate the association of each factor with the outcome. Our statistical analyses were completed using R version 4.1.3 (R Project for Statistical Computing).

### 2.6 Algorithms used in this study

In total, eight machine‐learning algorithms were used to create prediction models, including LR, Linear Discriminant Analysis (LDA), Random Forest (RF), Light Gradient Boosting machine (LightGBM), Gradient Boosting Machine (GBM), EXtreme Gradient Boosting (XGB), Support Vector Classifier (SVC), and Voting Ensemble (Voting).^26^, ^27^, ^28^, ^29^, ^30^, ^31^, ^32^, ^33^ A brief introduction to their parameter settings can be found in the Appendix S1.

Statistical methods have a long‐standing focus on inference, which is achieved through the creation and fitting of a project‐specific probability model. The model allows us to compute a quantitative measure of confidence that a discovered relationship describes a “true” effect that is unlikely to result from noise. By contrast, machine learning (ML) concentrates on prediction by using general‐purpose learning algorithms to find patterns in often rich and unwieldy data. ML methods are particularly helpful when one is dealing with “wide data”, where the number of input variables exceeds the number of subjects, in contrast to “long data”, where the number of subjects is greater than that of input variables. Classical statistics and ML vary in computational tractability as the number of variables per subject increases. Classical statistical modeling was designed for data with a few dozen input variables and sample sizes that would be considered small to moderate today. However, as the numbers of input variables and possible associations among them increase, the model that captures these relationships becomes more complex.^33^

### 2.7 Model training and testing

We included patient data from two sites, TMUH and WFH, in the training dataset. We conducted a stratified fivefold cross‐validation on the training set to evaluate the performance of different algorithms and errors. We divided all patients in the training dataset into five groups and assigned each group to be used as the internal validation set for one of five replications. After developing the training models, we used patient data from SHH for external testing to assess the model's generalization. The external testing demonstrated the ability of our model to predict outcomes from TMUH and WFH, as well as  future generalization to other hospitals.

### 2.8 Oversampling

We applied ADASYN (Adaptive Synthetic Sampling) resampling technique exclusively to the training dataset, while maintaining the original sample size of the testing dataset in alignment with real‐world clinical practice patterns. ADASYN represents a widely adopted approach within machine learning for addressing imbalanced datasets. This methodology strives to rectify class distribution imbalance by creating synthetic instances for the underrepresented class. A key feature of ADASYN involves its consideration of both data distribution and feature space density, thereby enhancing its effectiveness.^34^

### 2.9 Evaluation of model performance and interpretation

We computed various metrics, including the area under the receiver operating characteristic curve (AUROC), accuracy, sensitivity, specificity, positive predictive value (PPV), negative predictive value (NPV), and F1 score, to assess and contrast the performances of all prediction models. To determine the best model, we compared various models using the external test results and selected the model with the highest AUROC. We performed all data processing using MSSQL server 2017 and conducted model development and validation using Python programming language version 3.9.^35^ To interpret the model, we analyzed the influent levels of each predictor (i.e., feature importance) to the most optimal model applying SHapley Additive exPlanations (SHAP) values.^36^

## 3 RESULTS

Baseline characteristics of the study population, including patients' demographic information, diabetes treatment, comorbidities, long‐term medications, and laboratory test results, can be found in Table 1. The study encompassed records from 66,384 patients. The training dataset included 37,579 records and the test dataset included 28,805 records. Among all patients living with DM, 89 patients were diagnosed with pancreatic cancer. The mean age of all patients was 64.8 years, and the proportion of males (53.1%) was higher than that of females (46.9%). The mean BMI (26.2 kg/m^2^; SD: 4.87 kg/m^2^) was slightly above the healthy standard (18.5–24 kg/m^2^) at 26.2. The mean of the CCI score was 2.2 (SD: 1.43).

**TABLE 1:** Basic characteristics of the study cohort.

Feature	Overall (*N* = 66,384)	Training (*N* = 37,579)	Testing (*N* = 28,805)
Demographic			
Pancreatic cancer, no. (%)	89 (0.1%)	54 (0.1%)	35 (0.1%)
Age, years			
Mean (SD)	64.8 (12.2)	65.4 (12.4)	64.1 (11.9)
Median [Min, Max]	63.7 [45.1, 104]	64.4 [45.1, 104]	62.9 [45.1, 102]
Gender, no. (%)			
Female	31,108 (46.9%)	17,759 (47.3%)	13,349 (46.3%)
Male	35,278 (53.1%)	19,821 (52.7%)	15,457 (53.7%)
Body‐mass index (BMI), Mean (SD), kg/m^2^	26.2 (4.87)	26.2 (4.90)	26.3 (4.82)
Diabetes duration, years			
Mean (SD)	0.699 (1.74)	0.719 (1.78)	0.672 (1.69)
Anti‐diabetic agents, no. (%)			
Insulin	9364 (14.1%)	5469 (14.6%)	3895 (13.5%)
Biguanides	20,815 (31.4%)	11,990 (31.9%)	8825 (30.6%)
Sulfonylureas	4302 (6.5%)	2343 (6.2%)	1959 (6.8%)
Alpha glucosidase inhibitors	749 (1.1%)	416 (1.1%)	333 (1.2%)
Thiazolidinediones	180 (0.3%)	97 (0.3%)	83 (0.3%)
Dipeptidyl peptidase 4 (DPP‐4) inhibitors	2070 (3.1%)	1253 (3.3%)	817 (2.8%)
Glucagon‐like peptide‐1 (GLP‐1) analogues	23 (0.0%)	19 (0.1%)	4 (0.0%)
Sodium‐glucose co‐transporter 2 (SGLT2) inhibitors	136 (0.2%)	59 (0.2%)	77 (0.3%)
Other blood glucose‐lowering drugs, excl. insulin	1114 (1.7%)	565 (1.5%)	549 (1.9%)
Combined drugs	27,633 (41.6%)	15,369 (40.9%)	12,264 (42.6%)
Comorbidities			
Cardiovascular diseases, no. (%)	4715 (7.1%)	3345 (8.9%)	1370 (4.8%)
Chronic obstructive pulmonary disease (COPD), no. (%)	1321 (2.0%)	1129 (3.0%)	192 (0.7%)
Rheumatic, no. (%)	134 (0.2%)	96 (0.3%)	38 (0.1%)
Peptic ulcer disease, no. (%)	1372 (2.1%)	1141 (3.0%)	231 (0.8%)
Paralysis, no. (%)	33 (0.0%)	29 (0.1%)	4 (0.0%)
Renal disease, no. (%)	2008 (3.0%)	1297 (3.5%)	711 (2.5%)
Liver disease, no. (%)	3452 (5.2%)	2854 (7.6%)	598 (2.1%)
Anemias, no. (%)	959 (1.4%)	766 (2.0%)	193 (0.7%)
Depression, no. (%)	1594 (2.4%)	1328 (3.5%)	266 (0.9%)
Hyperlipidemia, no. (%)	14,821 (22.3%)	10,669 (28.4%)	4152 (14.4%)
Hypertension, no. (%)	17,493 (26.4%)	12,001 (31.9%)	5492 (19.1%)
Parkinson, no. (%)	269 (0.4%)	194 (0.5%)	75 (0.3%)
Prior stroke, no (%)	1981 (3.0%)	1134 (3.0%)	847 (2.9%)
CCI_score			
Mean (SD)	2.20 (1.43)	2.32 (1.49)	2.05 (1.33)
Median (Min, Max)	2.00 [0, 12.0]	2.00 [0, 12.0]	2.00 [0, 11.0]
Long‐term medications (ATC), *N* (%)			
Antacids (A02AA, A02AX)	1000 (1.5%)	817 (2.2%)	183 (0.6%)
Drugs for peptic ulcer and gastro‐esophageal reflux disease (A02BA, A02BC)	596 (0.9%)	369 (1.0%)	227 (0.8%)
Gastrointestinal disorders (A03AX, A03FA)	555 (0.8%)	439 (1.2%)	116 (0.4%)
Laxatives (A06AB, A06AD)	1524 (2.3%)	1212 (3.2%)	312 (1.1%)
Antithrombotic (B01AA, B01AC)	5251 (7.9%)	3834 (10.2%)	1417 (4.9%)
Antianemic agents (B03BA, B03BB, B03XA)	1036 (1.6%)	720 (1.9%)	316 (1.1%)
Cardiac therapy (C01AA, C01BD, C01DA, C01DX)	2109 (3.2%)	1486 (4.0%)	623 (2.2%)
Antihypertensives (C02CA, C02DB)	413 (0.6%)	290 (0.8%)	123 (0.4%)
Diuretics (C03AA, C03BA, C03CA, C03DA)	2599 (3.9%)	2065 (5.5%)	534 (1.9%)
Beta blocking agents (C07AA, C07AB, C07AG)	4591 (6.9%)	3406 (9.1%)	1185 (4.1%)
Calcium channel blockers (C08CA, C08DB)	4509 (6.8%)	3527 (9.4%)	982 (3.4%)
Renin angiotensin (C09AA, C09CA, C09DB, C09DX)	6815 (10.3%)	5209 (13.9%)	1606 (5.6%)
Lipid modifying agents (C10AA, C10AB, C10AX, C10BA)	7439 (11.2%)	5473 (14.6%)	1966 (6.8%)
Antiinflammatory and antirheumatic, non‐steroids (M01AB, M01AC, M01AH)	500 (0.8%)	358 (1.0%)	142 (0.5%)
Antigout (M04AA, M04AB, M04AC)	1403 (2.1%)	1144 (3.0%)	259 (0.9%)
Nervous system (N02AJ, N02BE, N03AE, N03AX, N04BA, N05AH, N05BA, N05BB, N05CD, N05CF, N06AA, N06AX, N06BX, N07AB, N07CA)	3518 (5.3%)	2599 (6.9%)	919 (3.2%)
Antihistamines (R06AE, R06AX)	323 (0.5%)	232 (0.6%)	91 (0.3%)
Peripheral vasodilators (C04AD)	803 (1.2%)	506 (1.3%)	297 (1.0%)
Liver therapy (A05BA)	430 (0.6%)	310 (0.8%)	120 (0.4%)
Alpha‐adrenoreceptor antagonists (G04CA)	507 (0.8%)	419 (1.1%)	88 (0.3%)
Glucocorticoids (H02AB)	150 (0.2%)	109 (0.3%)	41 (0.1%)
Thyroid hormones (H03AA)	355 (0.5%)	272 (0.7%)	83 (0.3%)
Laboratory tests			
HbA1c (glycated hemoglobin), (%)			
Mean (SD)	8.04 (2.01)	7.88 (1.87)	8.26 (2.17)
Median (min, max)	7.40 (3.40, 20.4)	7.30 (3.40, 19.1)	7.60 (4.00, 20.4)
Glucose AC, (mg/dL)			
Mean (SD)	160 (75.1)	158 (80.7)	163 (66.9)
Median (min, max)	139 (20.0, 1480)	136 (20.0, 1480)	143 (29.0, 1050)
Creatinine, (mg/dL)			
Mean (SD)	1.15 (1.19)	1.14 (1.14)	1.15 (1.25)
Median (min, max)	0.890 (0.0100, 23.7)	0.900 (0.200, 23.7)	0.860 (0.0100, 21.5)
Triglycerides, (mg/dL)			
Mean (SD)	171 (199)	162 (157)	181 (242)
Median (min, max)	134 (11.0, 8290)	130 (18.0, 6330)	138 (11.0, 8290)
Total cholesterol, (mg/dL)			
Mean (SD)	189 (45.4)	186 (43.1)	194 (47.8)
Median (min, max)	185 (66.0, 986)	181 (66.0, 815)	190 (66.0, 986)

Abbreviations: CCI, Charlson comorbidity index; SD, standard deviation; min, minimum; max, maximum.

Table 2 shows the results of both univariate and multivariate of Logistic Regression analysis. Prior to adjustment, patients with documented male sex, hypertension comorbidity, higher CCI score, laxative users, diuretic users, higher HbA1c, or higher AC glucose were significantly associated with a higher risk for pancreatic cancer. Conversely, patients who used biguanides and combinations of oral blood glucose‐lowering drugs were associated with a significantly lower risk of pancreatic cancer. After adjustment, only patients with hypertension, diuretic users, and those with higher HbA1c were correlated with a significantly higher risk of pancreatic cancer.

**TABLE 2:** Results of the logistic regression.

Feature	Univariate		Multivariate	
	Odds ratio (95% CI)	*p* value	Adj. odds ratio (95% CI)	*p* value
Demographic				
Gender	1.04 (1.02, 1.06)	**< 0.001**	0.996 (0.94, 1.05)	0.898
Age	0.83 (0.49, 1.42)	0.499	0.96 (0.55, 1.67)	0.895
Body‐mass index	0.91 (0.8, 1.03)	0.145	0.95 (0.85, 1.07)	0.384
Anti‐diabetic agent				
Reference = Insulin and analogues				
Biguanides	0.31 (0.15, 0.68)	**0.003**	0.48 (0.21, 1.09)	0.078
Sulfonylureas	0.44 (0.13, 1.5)	0.189	0.45 (0.13, 1.58)	0.212
Alpha glucosidase inhibitors	1.65 (0.38, 7.2)	0.506	1.68 (0.37, 7.6)	0.500
Thiazolidinediones	0 (0, Inf)	0.985	0 (0, Inf)	0.984
Dipeptidyl peptidase 4 (DPP‐4) inhibitors	0.27 (0.04, 2.06)	0.208	0.3 (0.04, 2.3)	0.246
Glucagon‐like peptide‐1 (GLP‐1) analogues	0 (0, Inf)	0.993	0 (0, Inf)	0.993
Sodium‐glucose co‐transporter 2 (SGLT2) inhibitors	0 (0, Inf)	0.988	0 (0, Inf)	0.988
Other blood glucose‐lowering drugs, excl. insulin	0.6 (0.08, 4.56)	0.625	0.53 (0.07, 4.09)	0.540
Combinations of oral blood glucose‐lowering drugs	0.44 (0.23, 0.86)	**0.016**	0.51 (0.25, 1.04)	0.064
Comorbidities				
Hyperlipidemia	0.88 (0.48, 1.62)	0.688	0.85 (0.39, 1.84)	0.671
Hypertension	1.98 (1.16, 3.38)	**0.012**	2.4 (1.22, 4.72)	**0.011**
Prior stroke	1.24 (0.3, 5.08)	0.768	0.61 (0.12, 3.14)	0.556
Cardiovascular diseases	2.05 (1, 4.2)	0.05	1.07 (0.35, 3.26)	0.907
Chronic obstructive pulmonary disease	1.9 (0.59, 6.1)	0.28	0.82 (0.23, 2.96)	0.765
Peptic ulcer disease	1.23 (0.3, 5.05)	0.775	0.54 (0.11, 2.74)	0.461
Renal disease	1.65 (0.51, 5.28)	0.402	0.57 (0.11, 3.04)	0.509
Liver disease	0.23 (0.03, 1.66)	0.145	0.17 (0.02, 1.35)	0.093
Depression	1.05 (0.26, 4.32)	0.946	0.74 (0.16, 3.33)	0.695
Charlson comorbidity index score	1.36 (1.17, 1.59)	**< 0.001**	1.43 (0.87, 2.34)	0.157
Long‐term medications				
Antacids	1.73 (0.42, 7.12)	0.446	0.71 (0.14, 3.64)	0.681
Gastro‐esophageal reflux disease	1.91 (0.26, 13.81)	0.524	1.22 (0.13, 11.08)	0.861
Gastrointestinal disorders	1.6 (0.22, 11.58)	0.643	0.68 (0.08, 5.96)	0.728
Laxatives	3.07 (1.22, 7.72)	**0.017**	1.73 (0.54, 5.55)	0.356
Antithrombotic	1.1 (0.47, 2.57)	0.825	0.52 (0.16, 1.66)	0.268
Antianemic	0.97 (0.13, 6.99)	0.973	0.54 (0.07, 4.51)	0.572
Cardiac therapy	2.48 (0.99, 6.24)	0.053	2.53 (0.76, 8.44)	0.132
Diuretics	3 (1.41, 6.36)	**0.004**	3.12 (1.11, 8.74)	**0.031**
Beta blockers	0.59 (0.18, 1.89)	0.374	0.26 (0.07, 1.02)	0.053
Calcium channel blockers	1.44 (0.65, 3.19)	0.37	1.07 (0.38, 3)	0.901
Renin angiotensin	1.08 (0.51, 2.29)	0.839	0.73 (0.25, 2.08)	0.555
Lipid‐modifying	0.87 (0.39, 1.93)	0.739	0.9 (0.3, 2.65)	0.845
Antigout	2.55 (0.92, 7.08)	0.072	2.79 (0.85, 9.17)	0.091
Nervous system drugs	1.37 (0.55, 3.45)	0.499	0.79 (0.26, 2.4)	0.676
Peripheral vasodilators	2.83 (0.69, 11.63)	0.15	2.01 (0.43, 9.37)	0.372
Thyroid	2.59 (0.36, 18.82)	0.346	1.67 (0.21, 13.63)	0.630
Laboratory tests				
HbA1c (glycated hemoglobin)	1.19 (1.03, 1.36)	**0.017**	1.22 (1.04, 1.42)	**0.013**
AC glucose	1.0 (1.0, 1.0048)	**0.004**	1.00 (0.9, 1.0048)	0.095
Creatinine	0.96 (0.67, 1.39)	0.833	0.61 (0.31, 1.2)	0.152
Triglyceride	0.99 (0.99, 1.00)	0.427	0.99 (0.99, 1.00)	0.497
Total cholesterol	0.99 (0.98, 1.00)	0.621	0.99 (0.98, 1.009)	0.743

Abbreviations: Adj., adjusted; CI, confidence interval.

**FIGURE 2:**
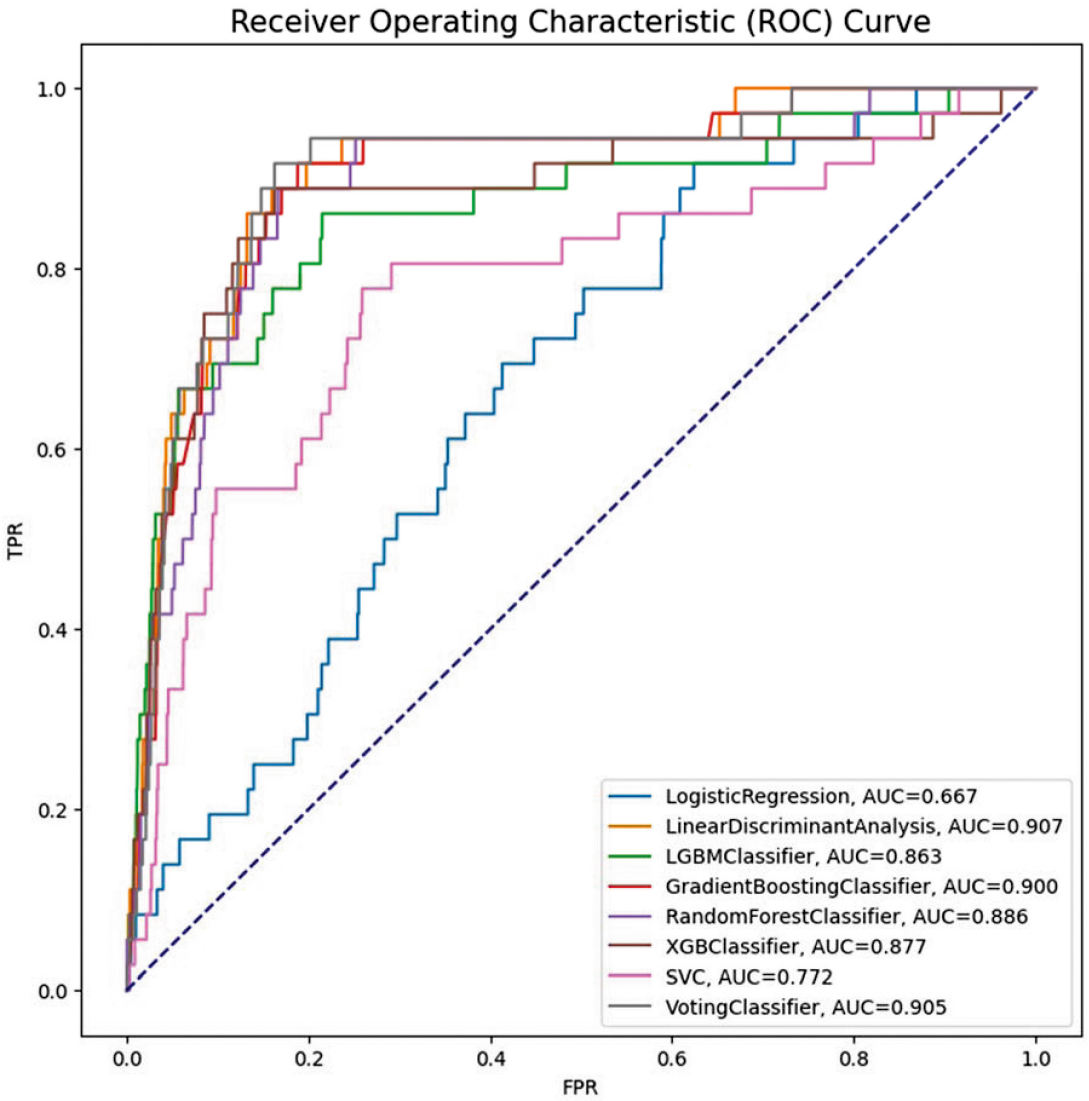
Receiver operating characteristics (ROC) curves of the prediction models.

The performance of the prediction models is shown in Table 3. The LDA model had a superior AUROC performance of 0.9073 compared to the other models: Voting (AUROC = 0.9049), GBM (AUROC = 0.9000), RF (AUROC = 0.8860), XGB (AUROC = 0.8772), LGBM (AUROC = 0.8632), SVC (AUROC = 0.7721), and LR (AUROC = 0.6669). The LDA model's other key performance measures were accuracy of 84.30%, sensitivity of 0.8611, and specificity of 0.8403. Respectively, the other models' accuracy, sensitivity, and specificity voting: 83.73%, 0.8889, and 0.8373; GBM: 81.20%, 0.8889, and 0.8120; RF: 83.36%, 0.8611, and 0.8336; XGB: 83.75%, 0.8611, and 0.8375; LGBM: 78.50%, 0.8333, and 0.7850; SVC: 74.09%, 0.7500, and 0.7409; and LR: 37.60%, 0.8889, and 0.3760. Figure 2 shows the AUROC values of various models in the four modes.

**TABLE 3:** Performance of the prediction models.

Model	Training‐AUROC	Testing‐AUROC	Accuracy	Sensitivity	Specificity	PPV	NPV	F1‐score
LR	0.7222	0.6669	0.3760	0.8889	0.3760	0.0001	1.0000	0.0002
LDA	0.9101	0.9073	0.8403	0.8611	0.8403	0.0002	1.0000	0.0012
LGBM	1.0000	0.8632	0.7850	0.8333	0.7850	0.0002	1.0000	0.0010
GBM	0.9103	0.9000	0.8120	0.8889	0.8120	0.0002	1.0000	0.0008
RF	0.9984	0.8860	0.8336	0.8611	0.8336	0.0002	1.0000	0.0015
XGB	0.9998	0.8772	0.8375	0.8611	0.8375	0.0002	1.0000	0.0009
SVC	0.7823	0.7721	0.7409	0.7500	0.7409	0.0001	1.0000	0.0003
Voting	0.9997	0.9049	0.8373	0.8889	0.8373	0.0002	1.0000	0.0009

Abbreviations: AUROC, area under the receiver operating characteristics curve; GBM, gradient boosting machine; LDA, linear discriminative analysis; LGBM, l gradient boosting machine; LR, logistic regression; NPV, negative predictive value; PPV, positive predictive value; RF, random forest; SVC, Support Vector Classifier; XGB, eXtreme Gradient Boosting.

**FIGURE 3:**
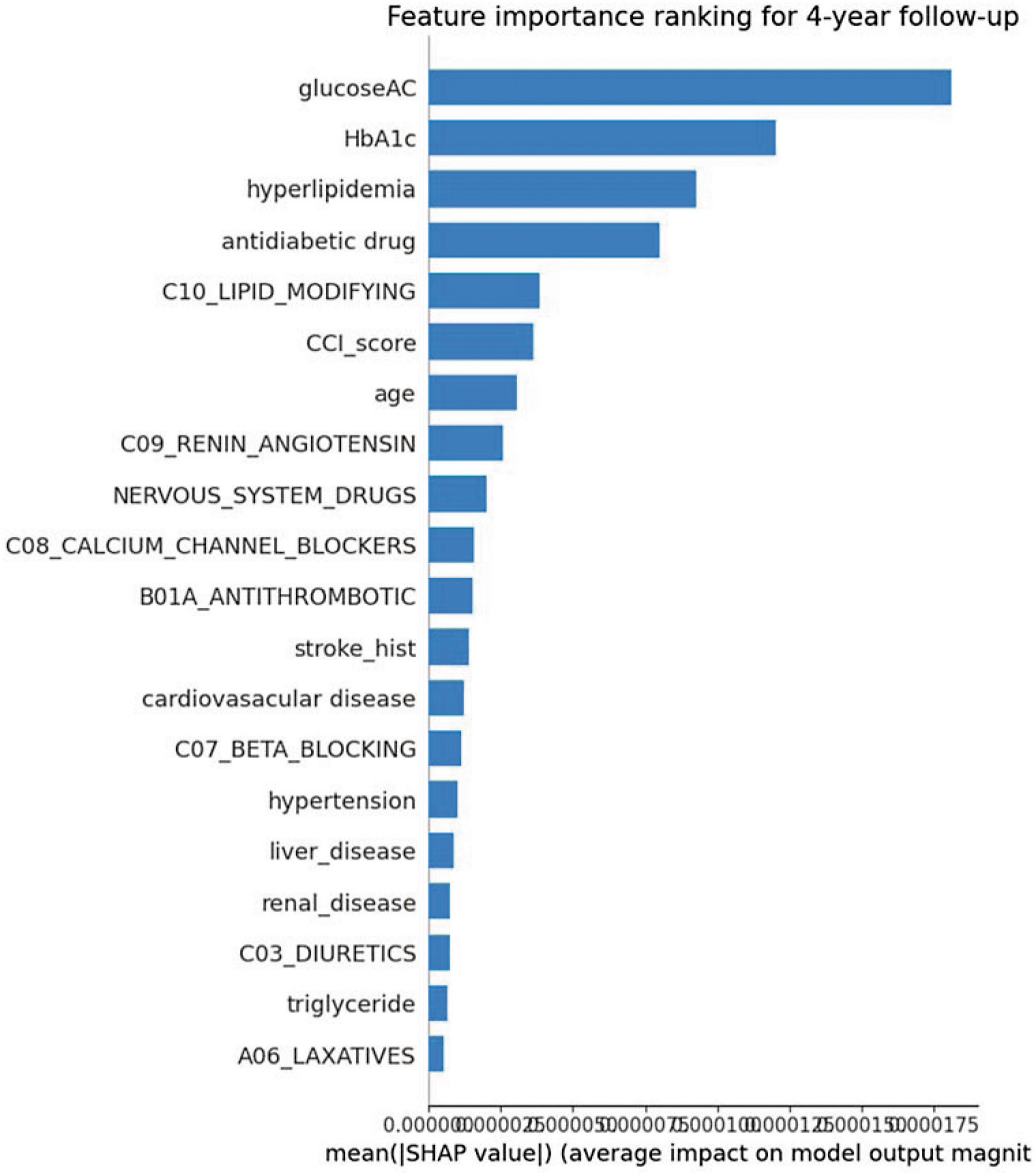
Importance ranking of features generated from the best model (linear discriminant analysis [LDA]).

Figure 3 shows LDA's feature importance levels of the model performance (in the full mode). The five most important features identified were glucose AC, HbA1c, hyperlipidemia comorbidity, antidiabetic drug use, and lipid‐modifying drug use.

## 4 DISCUSSION

Observational research is beneficial to early prevention interventions and disease detection as it offers a wide berth and depth of information on specific diseases and complications. In addition, emerging AI technologies afford an opportunity to establish personalized risk prediction models to optimize patient‐centered personalized medicine and outcomes. This pivot in contemporary clinical medicine allows researchers to go beyond traditional statistical methods of exploring relationships between variables in a population of individual patients. This is groundbreaking and supportive for rare disease prevention and management. It is of exceptional importance for patients with diabetes to engage in preventive measures and early detection to minimize future complications related to pancreatic cancer.^37^ Particularly, given the increased mortality rate of pancreatic cancer patients and the elevated risk of pancreatic cancer that diabetic populations experience.^37^ Previous studies conducted on the association between diabetes and the risk of pancreatic cancer identified a variety of potential risk factors. This study offers a more robust review by using more‐accurate AI prediction models. The continuous emergence of AI and sharing of information allows studies to build on each other. This study successfully used higher dimensional data and more advanced algorithms to construct a more‐accurate prediction model and explore the most vital predictors affecting the performance of the model. This can serve as a reference for future clinical decisions for treating diabetes and preventing pancreatic cancer.

Per previous publications, we found that higher HbA1c levels^17^, ^25^ which typically indicate poorly controlled diabetes, and patients with combidity of diabetes hypertension had a significantly higher risk of developing pancreatic cancer.^38^ Discordant from previous publications, this study found that diabetes patients who use diuretics for hypertension had a significantly higher risk of developing pancreatic cancer than those who do not use diuretics. This has has direct clinical implications for provider screenings and prevention education.

Besides the aforementioned risk factors, previous publications found that age,^18^, ^23^, ^24^ hepatitis B virus (HBV) status,^18^, ^39^, ^40^ and hepatobiliary malignancies were also risk factors for pancreatic cancer in patients with diabetes.^41^ However, this study did not find significant relationships for these factors. A number of previous studies showed that a higher BMI increases the risk of pancreatic cancer,^18^, ^38^, ^42^ while others indicated that a lower BMI increases the risk.^19^ This study did not find a suggestive association between BMI and pancreatic cancer pathophysiology. Finally, Baecker, et al. found that patients with diabetes and peptic ulcer disease had a higher risk of pancreatic cancer, while those with depression had a lower risk.^25^ However, these associations were not found to be significant in this study.

Using the TMUCRD provided this study several advantages over previous publications regarding data as a resource. First, previous studies mostly used older data, while this study used more‐recent clinical data (2009–2019). In addition, this study used a more comprehensive clinical predictor which sets it apart from previous research. Some previous studies did not include long‐term medication information, laboratory test results, or comorbidities and long‐term medication information.^2^, ^18^, ^23^ Whereas, we intentionally encompassed a wide range of data features, including demographic information; diabetes severity and treatments; comorbidities; long‐term medications; and laboratory test results based on updated literature reviews and clinical experts.

Constructing precise prediction models relies on multiple advanced AI algorithms. Most previous studies of risk prediction models for pancreatic cancer in patients with diabetes only used traditional bio‐statistical multivariate LR methods, or one AI algorithm to compare to the traditional LR method.^2^, ^18^, ^23^ The results of these studies do not show an advantage of AI algorithms over traditional LR methods.^2^, ^18^, ^23^ Due to the limitations of the algorithms produced, the predictive performance of previous studies on the risk prediction model for pancreatic cancer in patients with diabetes was lower than ours (AUROC values of <0.82). This study used eight algorithms (traditional LR and seven AI machine‐learning techniques: LDA, LightGBM, GBM, RF, XGB, SVC, and Voting). We compared the predictive performances of the models built with these different algorithms. He best prediction model in this study had an AUROC of 0.9073, which was higher than results of previous studies;^2^ this advantage is attributed to our use of multivariate algorithms and more comprehensive clinical data.

According to the best predictive model in this study, the two most significant predictive factors for the risk of pancreatic cancer were indicators of blood glucose control in diabetes (HbA1c and glucose AC). If blood glucose in diabetes is well controlled, the risk of pancreatic cancer can be greatly reduced, which is consistent with the results of most past observational studies and predictive model studies.^17^, ^23^, ^25^ Results of this study also showed that coexisting hyperlipidemia,as a comorbidity, was also a vital predictive factor for the development of pancreatic cancer in patients with diabetes. This aligns with the previous predictive model study (i.e., patients with diabetes and comorbid hyperlipidemia had a lower risk of developing pancreatic cancer).^23^

This study had several limitations. First, the study used electronic medical records from multiple hospitals as data sources, and although it included robust clinical information (i.e., demographics; disease treatment information; disease histories and comorbidities; long‐term medication use; and significant test results), it still lacked other less easily obtainable data types, such as personal lifestyle (i.e., diet, exercise, smoking, and drinking) and socioeconomic information. Future = research may collect this information and establish alternative prediction models. Second, the electronic medical records only contained detailed information on patients' services while visiting the TMUH, WFH, and SHH. Our research did not include information on visits to other medical facilities. Therefore, patients' clinical information might need to be more comprehensive, leading to inaccurate predictions in the model results. To address this concern and obtain a more accurate prediction model, this study adopted external validation mechanisms using data from two hospitals (TMUH and WFH) to establish the prediction model. Data from the third hospital (SHH) was used to conduct external tests to obtain the final prediction results. Finally, the data sources used in this study (including external validation) were all clinical data from Taiwanese hospitals. Prediction models established using data from a single country may lack generalizability for the results. Therefore, we suggest future research be carried out through international cooperation; using standardized case selection methods; the same research design and analytical methods; and data concepts following the protocol's guidance. This will allow enable using big data from different countries to understand differences among countries, achieve mutual verification of results, and enhance the clinical value of the prediction model.

## 5 CONCLUSIONS

This study successfully developed a novel and precise computer‐aided risk prediction model for diabetes complications and pancreatic cancer. Among various models, the LDA algorithm outperformed the highest AUROC (0.9073 by external testing). The significant clinical features that affected the model included glucose AC, HbA1c, hyperlipidemia as a comorbidity, antidiabetic drug use, and lipid‐modifying drug use. The model can support the clinical treatment and management of patients with diabetes to avoid pancreatic cancer. Clinicians might benefit from these results when considering patients' health conditions and prescribing medications.

## AUTHOR CONTRIBUTIONS

**Shih‐Min Chen:** Conceptualization (equal); investigation (equal); methodology (equal); resources (equal); writing – original draft (equal). **Thanh‐Phuc Phan:** Data curation (equal); formal analysis (equal); methodology (equal); visualization (equal); writing – original draft (equal). **Phung‐Anh Nguyen:** Data curation (equal); formal analysis (equal); software (equal). **Whitney Burton:** Writing – original draft (equal); writing – review and editing (equal). **Shwu‐Jiuan Lin:** Writing – original draft (equal). **Weei‐Chin Lin:** Writing – original draft (equal); writing – review and editing (equal). **Christine Y. Lu:** Writing – review and editing (equal). **Min‐Huei Hsu:** Supervision (supporting). **Chi‐Tsun Cheng:** Supervision (supporting); writing – review and editing (equal). **Jason C. Hsu:** Conceptualization (equal); funding acquisition (equal); investigation (equal); resources (equal); supervision (equal); writing – review and editing (equal).

## FUNDING INFORMATION

This work was supported by Taipei Medical University (grant number: TMU108‐AE1‐B42).

## CONFLICT OF INTEREST STATEMENT

The authors have no competing interests.

## ETHICS STATEMENT

This study was approved by the Taipei Medical University–Joint Institutional Review Board (TMU‐JIRB no.: N202101053). All data were anonymized and de‐identified before the analysis.

## Supporting information


Appendix S1.

## Data Availability

The authors obtained TMU clinical data from 2008 to 2020 (12 years) related to diabetes incidence in three medical centers from the TMUCRD.
