# Supplementary material for: A novel prediction model of the risk of pancreatic cancer among diabetes patients using multiple clinical data and machine learning
Source: Cancer Med. 2023 Sep 22;12(19):19987–99. doi: 10.1002/cam4.6547 (PMC10587954; doi:10.1002/cam4.6547)
Supplement: Supplementary file 1 — Appendix S1. [file CAM4-12-19987-s001.docx]

**A novel prediction model of the risk of pancreatic cancer among diabetes patients using multiple clinical data and machine learning**

**Supplementary Appendix**

**Machine learning algorithms**

Eight machine learning algorithms were used for model training and validation, including logistic regression (LR), Linear Discriminant Analysis (LDA), light gradient boosting machine (LightGBM), gradient boosting machine (GBM), random forest (RF), eXtreme gradient boosting (XGB), Support Vector Classifier (SVC), and Voting ensemble.

**(1) Logistic Regression (LR)**

Among the options in the latter category, one of the popular models in medicine is LR. An LR model calculates the class membership probability for one of two categories in a dataset.^1^ The parameters for modeling in this study were penalty = ‘l2’ and C = 0.0001.

**(2) Linear Discriminant Analysis (LDA)**

An LDA is generally used to classify patterns between two classes; however, it can be extended to classify multiple patterns. The LDA assumes that all classes are linearly separable, and according to this, multiple linear discrimination functions representing several hyperplanes in the feature space are created to distinguish the classes. If there are two classes, then the LDA draws one hyperplane and projects the data onto the hyperplane to maximize the separation of the two categories^2^. LDA parameters are established to solve for the recommended data with a large number of features using the least squares solution (lsqr), which can be combined with shrinkage or a custom covariance estimator with fixed shrinkage at 0 value.

**(3) Light Gradient Boosting Machine (LightGBM)**

The LightGBM is a gradient-boosting framework that uses tree-based learning algorithms. It was designed to be distributed and efficient with the following advantages: a faster training speed and higher efficiency, lower memory usage, better accuracy, support of parallel, distributed, and GPU learning, and capability to large-scale handling data^3^. The hyper-tuning parameters of the LGBM were as follows: objective = 'binary', class_weight = 'balanced', learning_rate = 0.05, reg_alpha = 0.1, reg_lambda = 0.1, subsample = 0.8, and random_state = 12.

**(4) Gradient Boosting Machine (GBM)**

Gradient boosting of regression trees produces competitive, highly robust, interpretable procedures for regression and classification. The ability of TreeBoost procedures to provide a quick indication of potential predictability, coupled with its extreme robustness, makes it a useful preprocessing tool that can be applied to imperfect data^4^. The GBM was applied with default parameters and n_estimators, which is the number of boosting stages to perform at 100.

**(5) Random Forest (RF)**

The RF fits many classification trees into a dataset and then combines predictions from all of the trees. The algorithm begins with the selection of bootstrap samples from the data. Observations in the original dataset that do not occur in a bootstrap sample are called out-of-bag observations. A classification tree fits each bootstrap sample, but at each node, only a small number of randomly selected variables are available for binary partitioning. The trees are fully grown, and each is used to predict the out-of-bag observations. The predicted observation class is calculated by the majority vote of the out-of-bag predictions for that observation, with ties randomly split^5^.

**(6) Extreme Gradient Boosting (XGB)**

XGBoost, an efficient and scalable implementation of the gradient boosting framework developed by Friedman, is a machine learning system for tree boosting. The scalability of XGBoost is due to several important systems and algorithmic optimizations. These innovations include a novel tree learning algorithm for handling sparse data; a theoretically justified weighted quantile sketch procedure enables handling instance weights in approximate tree learning. Parallel and distributed computing make learning faster, which enables quicker model exploration^6^.

**(7) Support Vector Classifier (SVC)**

The SVC is a machine-learning algorithm that can be applied to linear and nonlinear data. The SVC transforms original data to a higher dimension, from which it can use super vectors in the training dataset to find the hyperplane for categorizing the data. An SVC mainly identifies the hyperplane with the most significant margin, e.g., the maximum marginal hyperplane, to achieve higher accuracy^7^.

**(8) Voting ensemble**

Nilsson (1965) first proposed ensemble learning as a hybrid learning method for classification, combining several machine learning models in an intelligent way to produce predictions with greater accuracy and robustness than a single weak model. Through a methodology based on the variance component reduction of prediction errors of contributing models by adding bias, the efficacy acquired via ensemble method is accomplished^8^.

**Table 1. Basic Characteristics of the Study Cohort**

| **Feature** | **Overall  (*N*=66,384)** | **Training  (*N*=37,579)** | **Testing  (*N*=28,805)** | **P-value** |
| --- | --- | --- | --- | --- |
| **Demographic** |  |  |  |  |
| Pancreatic cancer, no. (%) | 89 (0.1%) | 54 (0.1%) | 35 (0.1%) |  |
| Age, years |  |  |  |  |
| Mean (SD) | 64.8 (12.2) | 65.4 (12.4) | 64.1 (11.9) | <0.001 |
| Median [Min, Max] | 63.7 [45.1, 104] | 64.4 [45.1, 104] | 62.9 [45.1, 102] |  |
| Gender, no. (%) |  |  |  |  |
| Female | 31,108 (46.9%) | 17,759 (47.3%) | 13,349 (46.3%) | 0.02 |
| Male | 35,278 (53.1%) | 19,821 (52.7%) | 15,457 (53.7%) |  |
| Body-mass index (BMI), kg/m^2^ |  |  |  |  |
| Mean (SD) | 26.2 (4.87) | 26.2 (4.90) | 26.3 (4.82) | 0.106 |
| Median [Min, Max] | 25.7 [12.7, 90.4] | 25.8 [12.7, 73.4] | 25.7 [12.8, 90.4] |  |
| Diabetes duration, years |  |  |  |  |
| Mean (SD) | 0.699 (1.74) | 0.719 (1.78) | 0.672 (1.69) | <0.001 |
| Anti-diabetic agents, no. (%) |  |  |  |  |
| Insulin | 9364 (14.1%) | 5469 (14.6%) | 3895 (13.5%) | <0.001 |
| Biguanides | 20,815 (31.4%) | 11,990 (31.9%) | 8825 (30.6%) |  |
| Sulfonylureas | 4302 (6.5%) | 2343 (6.2%) | 1959 (6.8%) |  |
| Alpha glucosidase inhibitors | 749 (1.1%) | 416 (1.1%) | 333 (1.2%) |  |
| Thiazolidinediones | 180 (0.3%) | 97 (0.3%) | 83 (0.3%) |  |
| Dipeptidyl peptidase 4 (DPP-4) inhibitors | 2070 (3.1%) | 1253 (3.3%) | 817 (2.8%) |  |
| Glucagon-like peptide-1 (GLP-1) analogues | 23 (0.0%) | 19 (0.1%) | 4 (0.0%) |  |
| Sodium-glucose co-transporter 2 (SGLT2) inhibitors | 136 (0.2%) | 59 (0.2%) | 77 (0.3%) |  |
| Other blood glucose-lowering drugs, excl. insulin | 1114 (1.7%) | 565 (1.5%) | 549 (1.9%) |  |
| Combined drugs | 27633 (41.6%) | 15369 (40.9%) | 12264 (42.6%) |  |
| **Comorbidities** |  |  |  |  |
| Cardiovascular diseases, no. (%) | 4715 (7.1%) | 3345 (8.9%) | 1370 (4.8%) | <0.001 |
| Chronic obstructive pulmonary disease (COPD), no. (%) | 1321 (2.0%) | 1129 (3.0%) | 192 (0.7%) | <0.001 |
| Rheumatic, no. (%) | 134 (0.2%) | 96 (0.3%) | 38 (0.1%) | <0.001 |
| Peptic ulcer disease, no. (%) | 1372 (2.1%) | 1141 (3.0%) | 231 (0.8%) | <0.001 |
| Paralysis, no. (%) | 33 (0.0%) | 29 (0.1%) | 4 (0.0%) | <0.001 |
| Renal disease, no. (%) | 2008 (3.0%) | 1297 (3.5%) | 711 (2.5%) | <0.001 |
| Liver disease, no. (%) | 3452 (5.2%) | 2854 (7.6%) | 598 (2.1%) | <0.001 |
| Anemias, no. (%) | 959 (1.4%) | 766 (2.0%) | 193 (0.7%) | <0.001 |
| Depression, no. (%) | 1594 (2.4%) | 1328 (3.5%) | 266 (0.9%) | <0.001 |
| Hyperlipidemia, no. (%) | 14,821 (22.3%) | 10,669 (28.4%) | 4152 (14.4%) | <0.001 |
| Hypertension, no. (%) | 17,493 (26.4%) | 12,001 (31.9%) | 5492 (19.1%) | <0.001 |
| Parkinson, no. (%) | 269 (0.4%) | 194 (0.5%) | 75 (0.3%) | <0.001 |
| Prior stroke, no (%) | 1981 (3.0%) | 1134 (3.0%) | 847 (2.9%) | 0.578 |
| CCI_score |  |  |  |  |
| Mean (SD) | 2.20 (1.43) | 2.32 (1.49) | 2.05 (1.33) | <0.001 |
| Median [Min, Max] | 2.00 [0, 12.0] | 2.00 [0, 12.0] | 2.00 [0, 11.0] |  |
| **Long-term medications (ATC), N (%)** |  |  |  |  |
| Antacids (A02AA, A02AX) | 1000 (1.5%) | 817 (2.2%) | 183 (0.6%) | <0.001 |
| Drugs for peptic ulcer and gastro-oesophageal reflux disease (A02BA, A02BC) | 596 (0.9%) | 369 (1.0%) | 227 (0.8%) | 0.00979 |
| Gastrointestinal disorders (A03AX, A03FA) | 555 (0.8%) | 439 (1.2%) | 116 (0.4%) | <0.001 |
| Laxatives (A06AB, A06AD) | 1524 (2.3%) | 1212 (3.2%) | 312 (1.1%) | <0.001 |
| Antithrombotic (B01AA, B01AC) | 5251 (7.9%) | 3834 (10.2%) | 1417 (4.9%) | <0.001 |
| Antianemic agents (B03BA, B03BB, B03XA) | 1036 (1.6%) | 720 (1.9%) | 316 (1.1%) | <0.001 |
| Cardiac therapy (C01AA, C01BD, C01DA, C01DX) | 2109 (3.2%) | 1486 (4.0%) | 623 (2.2%) | <0.001 |
| Antihypertensives (C02CA, C02DB) | 413 (0.6%) | 290 (0.8%) | 123 (0.4%) | <0.001 |
| Diuretics (C03AA, C03BA, C03CA, C03DA) | 2599 (3.9%) | 2065 (5.5%) | 534 (1.9%) | <0.001 |
| Beta blocking agents (C07AA, C07AB, C07AG) | 4591 (6.9%) | 3406 (9.1%) | 1185 (4.1%) | <0.001 |
| Calcium channel blockers (C08CA, C08DB) | 4509 (6.8%) | 3527 (9.4%) | 982 (3.4%) | <0.001 |
| Renin angiotensin (C09AA, C09CA, C09DB, C09DX) | 6815 (10.3%) | 5209 (13.9%) | 1606 (5.6%) | <0.001 |
| Lipid modifying agents (C10AA, C10AB, C10AX, C10BA) | 7439 (11.2%) | 5473 (14.6%) | 1966 (6.8%) | <0.001 |
| Anti-inflammatory and antirheumatic, non-steroids (M01AB, M01AC, M01AH) | 500 (0.8%) | 358 (1.0%) | 142 (0.5%) | <0.001 |
| Antigout (M04AA, M04AB, M04AC) | 1403 (2.1%) | 1144 (3.0%) | 259 (0.9%) | <0.001 |
| Nervous system (N02AJ, N02BE, N03AE, N03AX, N04BA, N05AH, N05BA, N05BB, N05CD, N05CF, N06AA, N06AX, N06BX, N07AB, N07CA) | 3518 (5.3%) | 2599 (6.9%) | 919 (3.2%) | <0.001 |
| Antihistamines (R06AE, R06AX) | 323 (0.5%) | 232 (0.6%) | 91 (0.3%) | <0.001 |
| Peripheral vasodilators (C04AD) | 803 (1.2%) | 506 (1.3%) | 297 (1.0%) | <0.001 |
| Liver therapy (A05BA) | 430 (0.6%) | 310 (0.8%) | 120 (0.4%) | <0.001 |
| Alpha-adrenoreceptor antagonists (G04CA) | 507 (0.8%) | 419 (1.1%) | 88 (0.3%) | <0.001 |
| Glucocorticoids (H02AB) | 150 (0.2%) | 109 (0.3%) | 41 (0.1%) | <0.001 |
| Thyroid hormones (H03AA) | 355 (0.5%) | 272 (0.7%) | 83 (0.3%) | <0.001 |
| **Laboratory Tests** |  |  |  |  |
| HbA1c (glycated hemoglobin), [%] |  |  |  |  |
| Mean (SD) | 8.04 (2.01) | 7.88 (1.87) | 8.26 (2.17) | <0.001 |
| Median [Min, Max] | 7.40 [3.40, 20.4] | 7.30 [3.40, 19.1] | 7.60 [4.00, 20.4] |  |
| Glucose AC, [mg/dl] |  |  |  |  |
| Mean (SD) | 160 (75.1) | 158 (80.7) | 163 (66.9) | <0.001 |
| Median [Min, Max] | 139 [20.0, 1480] | 136 [20.0, 1480] | 143 [29.0, 1050] |  |
| Creatinine, [mg/dl] |  |  |  |  |
| Mean (SD) | 1.15 (1.19) | 1.14 (1.14) | 1.15 (1.25) | 0.405 |
| Median [Min, Max] | 0.890 [0.01, 23.7] | 0.90 [0.20, 23.7] | 0.86 [0.01, 21.5] |  |
| Triglycerides, [mg/dl] |  |  |  |  |
| Mean (SD) | 171 (199) | 162 (157) | 181 (242) | <0.001 |
| Median [Min, Max] | 134 [11.0, 8290] | 130 [18.0, 6330] | 138 [11.0, 8290] |  |
| Total cholesterol, [mg/dl] |  |  |  |  |
| Mean (SD) | 189 (45.4) | 186 (43.1) | 194 (47.8) | <0.001 |
| Median [Min, Max] | 185 [66.0, 986] | 181 [66.0, 815] | 190 [66.0, 986] |  |

CCI, Charlson comorbidity index; SD, standard deviation; Min, minimum; Max, maximum.

**Table 2. Results of the Logistic Regression**

| **Feature** | **Univariate** | | **Multivariate** | |
| --- | --- | --- | --- | --- |
|  | **Odds ratio**  **(95% CI)** | ***p* value** | **Adj. odds ratio**  **(95% CI)** | ***p* value** |
| **Demographic** |  |  |  |  |
| Gender | 1.04 (1.02, 1.06) | **< 0.001** | 0.996 (0.94, 1.05) | 0.898 |
| Age | 0.83 (0.49, 1.42) | 0.499 | 0.96 (0.55, 1.67) | 0.895 |
| Body-mass index | 0.91 (0.8, 1.03) | 0.145 | 0.95 (0.85, 1.07) | 0.384 |
| **Anti-diabetic agent**  Reference = Insulin and analogues |  |  |  |  |
| Biguanides | 0.31 (0.15, 0.68) | **0.003** | 0.48 (0.21, 1.09) | 0.078 |
| Sulfonylureas | 0.44 (0.13, 1.5) | 0.189 | 0.45 (0.13, 1.58) | 0.212 |
| Alpha glucosidase inhibitors | 1.65 (0.38, 7.2) | 0.506 | 1.68 (0.37, 7.6) | 0.500 |
| Thiazolidinediones | 0 (0, Inf) | 0.985 | 0 (0, Inf) | 0.984 |
| Dipeptidyl peptidase 4 (DPP-4) inhibitors | 0.27 (0.04, 2.06) | 0.208 | 0.3 (0.04, 2.3) | 0.246 |
| Glucagon-like peptide-1 (GLP-1) analogues | 0 (0, Inf) | 0.993 | 0 (0, Inf) | 0.993 |
| Sodium-glucose co-transporter 2 (SGLT2) inhibitors | 0 (0, Inf) | 0.988 | 0 (0, Inf) | 0.988 |
| Other blood glucose-lowering drugs, excl. insulin | 0.6 (0.08, 4.56) | 0.625 | 0.53 (0.07, 4.09) | 0.540 |
| Combinations of oral blood glucose-lowering drugs | 0.44 (0.23, 0.86) | **0.016** | 0.51 (0.25, 1.04) | 0.064 |
| **Comorbidities** |  |  |  |  |
| Hyperlipidemia | 0.88 (0.48, 1.62) | 0.688 | 0.85 (0.39, 1.84) | 0.671 |
| Hypertension | 1.98 (1.16, 3.38) | **0.012** | 2.4 (1.22, 4.72) | **0.011** |
| Prior stroke | 1.24 (0.3, 5.08) | 0.768 | 0.61 (0.12, 3.14) | 0.556 |
| Cardiovascular diseases | 2.05 (1, 4.2) | 0.05 | 1.07 (0.35, 3.26) | 0.907 |
| Chronic obstructive pulmonary disease | 1.9 (0.59, 6.1) | 0.28 | 0.82 (0.23, 2.96) | 0.765 |
| Peptic ulcer disease | 1.23 (0.3, 5.05) | 0.775 | 0.54 (0.11, 2.74) | 0.461 |
| Renal disease | 1.65 (0.51, 5.28) | 0.402 | 0.57 (0.11, 3.04) | 0.509 |
| Liver disease | 0.23 (0.03, 1.66) | 0.145 | 0.17 (0.02, 1.35) | 0.093 |
| Depression | 1.05 (0.26, 4.32) | 0.946 | 0.74 (0.16, 3.33) | 0.695 |
| Charlson comorbidity index score | 1.36 (1.17, 1.59) | **< 0.001** | 1.43 (0.87, 2.34) | 0.157 |
| **Long-term medications** |  |  |  |  |
| Antacids | 1.73 (0.42, 7.12) | 0.446 | 0.71 (0.14, 3.64) | 0.681 |
| Gastro-esophageal reflux disease | 1.91 (0.26, 13.81) | 0.524 | 1.22 (0.13, 11.08) | 0.861 |
| Gastrointestinal disorders | 1.6 (0.22, 11.58) | 0.643 | 0.68 (0.08, 5.96) | 0.728 |
| Laxatives | 3.07 (1.22, 7.72) | **0.017** | 1.73 (0.54, 5.55) | 0.356 |
| Antithrombotic | 1.1 (0.47, 2.57) | 0.825 | 0.52 (0.16, 1.66) | 0.268 |
| Antianemic | 0.97 (0.13, 6.99) | 0.973 | 0.54 (0.07, 4.51) | 0.572 |
| Cardiac therapy | 2.48 (0.99, 6.24) | 0.053 | 2.53 (0.76, 8.44) | 0.132 |
| Diuretics | 3 (1.41, 6.36) | **0.004** | 3.12 (1.11, 8.74) | **0.031** |
| Beta blockers | 0.59 (0.18, 1.89) | 0.374 | 0.26 (0.07, 1.02) | 0.053 |
| Calcium channel blockers | 1.44 (0.65, 3.19) | 0.37 | 1.07 (0.38, 3) | 0.901 |
| Renin angiotensin | 1.08 (0.51, 2.29) | 0.839 | 0.73 (0.25, 2.08) | 0.555 |
| Lipid-modifying | 0.87 (0.39, 1.93) | 0.739 | 0.9 (0.3, 2.65) | 0.845 |
| Antigout | 2.55 (0.92, 7.08) | 0.072 | 2.79 (0.85, 9.17) | 0.091 |
| Nervous system drugs | 1.37 (0.55, 3.45) | 0.499 | 0.79 (0.26, 2.4) | 0.676 |
| Peripheral vasodilators | 2.83 (0.69, 11.63) | 0.15 | 2.01 (0.43, 9.37) | 0.372 |
| Thyroid | 2.59 (0.36, 18.82) | 0.346 | 1.67 (0.21, 13.63) | 0.630 |
| **Laboratory tests** |  |  |  |  |
| HbA1c (glycated hemoglobin) | 1.19 (1.03, 1.36) | **0.017** | 1.22 (1.04, 1.42) | **0.013** |
| A... (AC) glucose | 1.0 (1.0, 1.0048) | **0.004** | 1.00 (0.9, 1.0048) | 0.095 |
| Creatinine | 0.96 (0.67, 1.39) | 0.833 | 0.61 (0.31, 1.2) | 0.152 |
| Triglyceride | 0.99 (0.99, 1.00) | 0.427 | 0.99 (0.99, 1.00) | 0.497 |
| Total cholesterol | 0.99 (0.98, 1.00) | 0.621 | 0.99 (0.98, 1.009) | 0.743 |

CI, confidence interval; Adj., adjusted.

**References**

1. Dreiseitl S, Ohno-Machado L. Logistic regression and artificial neural network classification models: A methodology review. *Journal of Biomedical Informatics.* 2002;35:352-359.

2. Vaibhaw, Sarraf J, Pattnaik PK. Brain-computer interfaces and their applications. *An Industrial IoT Approach for Pharmaceutical Industry Growth: Volume 2.* 2020:31-54.

3. Microsoft C. *LightGBM.* 2022.

4. Friedman JH. Greedy Function Approximation: A Gradient Boosting Machine. *The Annals of Statistics.* 2001;29:1189-1232.

5. Cutler DR, Edwards TC, Beard KH, et al. Random forests for classification in ecology. *Ecology.* 2007;88:2783-2792.

6. Chen T, Guestrin C. XGBoost: A Scalable Tree Boosting System. *22nd ACM SIGKDD International Conference on Knowledge Discovery and Data Mining.* 2016:785-794.

7. Gunn, S.R. Support vector machines for classification and regression. ISIS Tech. Rep. 1998, 14, 5–16.

8. Kibria HB, Nahiduzzaman M, Goni MOF, Ahsan M, Haider J. An Ensemble Approach for the Prediction of Diabetes Mellitus Using a Soft Voting Classifier with an Explainable AI. *Sensors.* 2022;22(19):7268.
